# A Novel CYP2E1 Inhibitor, 4‐Methyl‐5‐Acetylthiazole (Q11), Alleviates Obesity Via Modulating Adipose Inflammation and Mitochondrial Dysfunction

**DOI:** 10.1002/advs.202515315

**Published:** 2025-12-20

**Authors:** Jinhuan Qiu, Liyuan Gao, Liyang Wang, Xueke Wang, Lin Jia, Mengyan Deng, Liming Tang, Qiang Wen, Na Gao, Haiwei Xu, Hailing Qiao

**Affiliations:** ^1^ Institute of Clinical Pharmacology School of Basic Medical Sciences Zhengzhou University Zhengzhou China

**Keywords:** CYP2E1, obesity, Q11, inflammation, mitochondrial dysfunction, adipocyte

## Abstract

Obesity is a major global health challenge characterized by chronic low‐grade inflammation and impaired mitochondrial homeostasis. Although cytochrome P450 2E1 (CYP2E1) is implicated in oxidative stress and inflammatory signaling, its contribution to adipocyte dysfunction during obesity remains insufficiently defined. Here, we evaluate the functional role of CYP2E1 in obesity and the therapeutic potential of a highly selective CYP2E1 inhibitor, 4‐methyl‐5‐acetylthiazole (Q11). High‐fat diet‐induced obese mice exhibited markedly elevated CYP2E1 expression and activity, which positively correlated with increased adiposity, hepatic steatosis, and mitochondrial dysfunction. Pharmacological inhibition of CYP2E1 by Q11 significantly attenuated body weight gain, improved hepatic lipid accumulation, and reduced inflammatory responses without affecting food intake, suggesting that its metabolic benefits are mediated through enhanced energy expenditure. Mechanistically, Q11 restored mitochondrial integrity by increasing oxygen consumption, normalizing membrane potential, promoting mitochondrial biogenesis, and improving fusion dynamics, accompanied by activation of the AMP‐activated protein kinase/peroxisome proliferator‐activated receptor‐gamma coactivator 1‐alpha pathway. Collectively, these findings identify CYP2E1 as a previously unrecognized regulator of obesity‐associated metabolic dysfunction and establish Q11 as a promising therapeutic candidate that concurrently suppresses inflammation and reinstates mitochondrial homeostasis. This work provides a mechanistic and translational foundation for targeting CYP2E1 in obesity and related metabolic disorders.

## Graphical abstract

Obesity involves chronic inflammation and mitochondrial dysfunction. This study identifies cytochrome P450 2E1 (CYP2E1) as a novel inflammatory target in adipose tissue. Its activity increases in obese mice and correlates with inflammation and mitochondrial impairment. Pharmacological CYP2E1 inhibition with 4‐methyl‐5‐acetylthiazole (Q11) reduces weight gain, alleviates adipose inflammation, and restores mitochondrial function via AMPK/PGC‐1α signaling, presenting a promising therapeutic strategy for obesity.

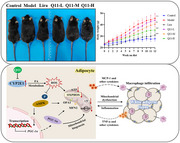

## 1 Introduction

Obesity is a chronic metabolic disease characterized by adipose tissue dysfunction and systemic metabolic derangements [1, 2]. Mounting evidence indicates that chronic low‐grade inflammation and impaired mitochondrial homeostasis within white adipose tissue (WAT) are central drivers of obesity‐related complications, including type 2 diabetes mellitus (T2DM), nonalcoholic fatty liver disease (NAFLD), cardiovascular disorders, and several malignancies [3, 4, 5, 6, 7]. These pathogenic processes highlight the urgent need for strategies that restore adipose metabolic integrity.

Current pharmacological options, such as glucagon‐like peptide‐1 (GLP‐1) receptor agonists, achieve meaningful weight reduction but suffer from high cost, tolerability issues, and long‐term safety concerns [8, 9, 10]. This underscores the need for novel, safe, and effective therapeutic options.

Cytochrome P450 2E1 (CYP2E1), a major member of the cytochrome P450 superfamily, participates in the metabolism of various endogenous and exogenous substrates [11]. Beyond its canonical metabolic role, CYP2E1 has emerged as a pivotal contributor to inflammatory pathogenesis by promoting excessive reactive oxygen species (ROS) generation and pro‐inflammatory cytokine production (e.g., TNF‐α, IL‐6) [12, 13]. Elevated CYP2E1 expression and activity have been implicated in multiple chronic disorders, including alcoholic steatohepatitis, NAFLD, T2DM, obesity, and cancer [14, 15, 16, 17, 18, 19, 20, 21, 22, 23]. Our previous studies revealed that CYP2E1 upregulation correlates with disease severity across diverse inflammatory conditions, such as sepsis, rheumatoid arthritis, severe acute pancreatitis, and malignancies [21, 22, 23, 24, 25, 26, 27]. Building on these insights, we developed a novel, selective CYP2E1 inhibitor, 4‐methyl‐5‐acetylthiazole (Q11), which demonstrated favorable efficacy, safety, and druggability in preclinical models of inflammation [21, 22, 23, 24, 25, 26, 27].

Although CYP2E1 has been associated with obesity, existing evidence remains limited and largely correlative. Clinical studies report elevated CYP2E1 activity in obese individuals, which normalizes after bariatric surgery and associates with hepatic steatosis [16, 28, 29, 30, 31]. In rodents, *Cyp2e1* deletion confers resistance to high‐fat diet (HFD)‐induced obesity and metabolic dysfunction [32, 33]. At the cellular level, a CYP2E1⁺ALDH1A1⁺ adipocyte subset has been implicated in thermogenesis, while increased CYP2E1 expression modulates lipid metabolism in adipose progenitors [34, 35]. These observations hint at a role for CYP2E1 in adipose energy regulation. However, whether CYP2E1 actively drives adipocyte inflammation and mitochondrial dysfunction in obesity—and the underlying mechanisms—remains unknown.

Here, we demonstrate that CYP2E1 activity is markedly elevated in HFD‐induced obese mice and positively correlates with adiposity and adipocyte dysfunction. Basal CYP2E1 activity further predicts susceptibility to diet‐induced weight gain. Pharmacological inhibition of CYP2E1 with Q11 confers robust protection against obesity and metabolic impairment in HFD‐induced obese mice. Mechanistic studies integrating transcriptomic profiling, in vivo metabolic phenotyping, and in vitro analyses reveal that Q11 alleviates adipocyte inflammation and preserves mitochondrial function via activation of the AMP‐activated protein kinase (AMPK)–peroxisome proliferator‐activated receptor‐gamma coactivator 1‐alpha (PGC‐1α) axis. Collectively, these findings uncover an unrecognized role for CYP2E1 in adipose metabolic homeostasis and establish Q11 as a promising therapeutic candidate for obesity and its associated complications.

## 2 Methods

### 2.1 Chemicals and Reagents

Q11 (purity > 98%), 4‐methyl‐5‐acetylthiazole, was synthesized by our laboratory, and its chemical structure is provided in (Figure S1). 3‐Isobutyl‐1‐methylxanthine (IBMX) and palmitic acid (PA) were purchased from Sigma–Aldrich (USA). Recombinant human insulin injection was obtained from Novo Nordisk (Denmark). Dexamethasone, rosiglitazone, and Compound C were purchased from Shanghai MCE Co., Ltd. Recombinant human TNF‐α protein was acquired from Abcam (UK).

### 2.2 Animals and Treatments

Male C57BL/6J mice (6 weeks old) were housed in a specific pathogen‐free facility under a 12 h light/dark cycle with ad libitum access to food and water. After one week of acclimation, mice were randomly divided into six experimental groups (*n* = 10 per group). The control group received a standard low‐fat, low‐sugar diet (TP23302; Nantong Trophic Animal Feed High‐Tech Co., Ltd.) along with daily intragastric administration of sterile saline. The remaining groups were maintained on a HFD providing 60% of calories from fat  (TP23300) for the duration of the study. Among these, the model group received saline, while the positive control group was treated with liraglutide (Lira, 200 µg/kg/day) via subcutaneous injection. The other three groups received Q11—freshly dissolved in sterile saline—at low (Q11‐L, 15 mg/kg/day), medium (Q11‐M, 30 mg/kg/day), or high (Q11‐H, 60 mg/kg/day) doses via daily intragastric gavage. All interventions commenced simultaneously with the initiation of the high‐fat diet and continued for 12 weeks. The animal study protocol was approved by the Institutional Animal Care and Use Committee of Zhengzhou University (Approval No. ZZUIRB2022–152).

### 2.3 in vivo Assessment of CYP2E1 Enzymatic Activity

CYP2E1 activity in vivo was quantified by pharmacokinetic analysis of its probe substrate chlorzoxazone (CZX). After overnight fasting, mice were injected with 60 mg/kg CZX via the tail vein. Blood was collected 15 min post‐injection from the orbital venous plexus into heparinized tubes, and plasma was separated by centrifugation. Levels of CZX and its metabolite 6‐OH chlorzoxazone (6‐OH CZX) were determined by high‐performance liquid chromatography (HPLC) using a Kromasil 100‐5‐C18 column (4.6 × 250 mm) at 30°C. The mobile phase was methanol:water (56:44, v/v) at 1.0 mL/min. Detection wavelength was 287 nm. CYP2E1 activity was expressed as the 6‐OH CZX/CZX ratio at 15 min post‐injection.

### 2.4 Body Composition Analysis

Body composition of awake mice was measured using a quantitative magnetic resonance system (QMR12‐060H‐I, Suzhou Nuomai, China).

### 2.5 Metabolic Cage Studies

Energy metabolism parameters, including oxygen consumption (VO_2_), carbon dioxide production (VCO_2_), and energy expenditure (EE) were continuously monitored using an eight‐chamber PhenoMaster system (TSE Systems, Germany).

### 2.6 Serum Lipid Profiles and Liver Enzymes Analyses

Serum levels of total cholesterol (TC), low‐density lipoprotein cholesterol (LDL‐C), alanine aminotransferase (ALT), and aspartate aminotransferase (AST) were measured using commercial kits from Nanjing Jiancheng Bioengineering Institute according to the manufacturer's instructions (Cat# A111‐1‐1, A113‐1‐1, C009‐2‐1, and C010‐2‐1, respectively).

### 2.7 Serum Adipokines and Cytokines Measurement

Serum concentrations of adiponectin (ADIPOQ), monocyte chemoattractant protein‐1 (MCP‐1), TNF‐α, and IL‐1β were measured using ELISA kits (Fine Biotech, Wuhan, China; Cat# EM0001, EM0135‐HS, EM0183‐HS, QT‐EM0109).

### 2.8 Hepatic Triglyceride (TG) Content

Hepatic TG content was quantified using a high‐fat tissue TG detection kit (Precilab, Beijing, China; Cat# E1025).

### 2.9 Transcriptome Analysis

Total RNA was extracted from epididymal white adipose tissue (eWAT) and subjected to RNA sequencing. Differential gene expression and pathway enrichment analyses were performed using the Bioinformatics Cloud Platform (Wuhan Maiwei Metabolism Co., LTD).

### 2.10 Electron Microscopy of Adipose Tissue

Freshly dissected adipose tissues were immediately fixed in 2% paraformaldehyde and 2.5% glutaraldehyde in 0.15 m sodium cacodylate buffer (pH 7.4) overnight at 4°C. Samples were post‐fixed in 1% osmium tetroxide, dehydrated through a graded ethanol series, and embedded in Durcupan resin. Ultrathin sections (60 nm) were cut using a Leica microtome with a diamond knife, stained with uranyl acetate and lead citrate, and examined by transmission electron microscopy.

### 2.11 Oxidative Stress Marker Assays

Levels of reduced glutathione (GSH) and malondialdehyde (MDA) in eWAT were determined using kits from Nuominkeda Biotechnology (Wuhan, China; Cat# NM‐W‐0407 and NM‐W‐0104).

### 2.12 Primary Adipocyte Isolation

At sacrifice, eWAT and inguinal white adipose tissue (iWAT) were collected from mice (*n* = 3/group), minced, and digested with 1 mg/mL collagenase type I at 37°C for 15–20 min (eWAT) or 20–30 min (iWAT) with gentle shaking. Digestion was quenched with high‐glucose Dulbecco's Modified Eagle Medium (DMEM) plus 10% serum, filtered through a 100 µm strainer, and centrifuged at 200 × g for 3 min to separate mature adipocytes. Adipocytes were washed twice with PBS and pelleted at 1000 rpm for downstream analyses.

### 2.13 3T3‐L1 Adipocyte Differentiation

3T3‐L1 preadipocytes (Shanghai Cell Bank, Chinese Academy of Sciences) were cultured in high‐glucose DMEM supplemented with 10% newborn calf serum. Two days post‐confluence, differentiation was induced by treating cells with 0.5 mm IBMX, 5 µm dexamethasone, 2 µm rosiglitazone, and 2 µg/mL insulin for 4 days, followed by insulin‐only medium (days 4–7) and maintenance in growth medium thereafter (> 90% lipid accumulation).

### 2.14 Adipokine and MCP‐1 Secretion Assay

Differentiated 3T3‐L1 adipocytes were treated under control conditions, PA (400 µm) + TNF‐α (10 ng/mL), or model + Q11 (12.5, 25, 50 µm) for 48 h. Supernatants were collected for ELISA measurement of ADIPOQ and MCP‐1 (Elabscience; Cat# E‐EL‐M0002, E‐EL‐M3001).

### 2.15 Intracellular ROS Measurement

3T3‐L1 adipocytes were treated as described above, then incubated with 10 µm DCFH‐DA probe at 37°C for 25 min. Cells were washed and analyzed by flow cytometry for ROS levels using FlowJo software.

### 2.16 Mitochondrial Membrane Potential (ΔΨm) Assay

JC‐1 staining was performed following treatment to assess ΔΨm. Cells were incubated with JC‐1 working solution at 37°C for 20 min, washed, and analyzed by flow cytometry.

### 2.17 Oxygen Consumption Rate (OCR) Measurement

Differentiated 3T3‐L1 adipocytes were seeded in black clear‐bottom 96‐well plates at 1 × 10⁶ cells/mL and treated as described. OCR was measured using a fluorescence‐based oxygen consumption assay kit (Elabscience, E‐BC‐F068) on a PerkinElmer microplate reader, with kinetic readings every 2 min over 90 min.

### 2.18 ATP Content Measurement

After treatment, 2 × 10⁶ cells were lysed by boiling in assay reagent and centrifuged. ATP levels in supernatants were quantified using a luminescence‐based ATP assay kit on a chemiluminescence microplate reader (PerkinElmer).

### 2.19 Western Blotting

Proteins were extracted using RIPA buffer (Solarbio, China). Concentrations were measured using a BCA assay (GlbBio, USA). Sodium dodecyl sulfate–polyacrylamide gel electrophoresis resolved equal amounts, transferred to polyvinylidene fluoride membranes (Millipore, USA), blocked in 5% bovine serum albumin, and incubated overnight at 4°C with primary antibodies including CYP2E1, MFN2 (HUABIO, #R1511‐7, HA720073), COXIV and β‐actin (Proteintech, #11242‐1‐AP, #66009‐1‐Ig), SDHB (Zenbio, #381845), NDUFB8, UQCRC2, MTCO1, ATP5A AMPK, p‐AMPK, PGC‐1α, TFAM, OPA1, DRP1, β‐tubulin (Signalway Antibody, #49897, #56271, #30224, #40628, #21191, #11183, #27583, #29688, #49680, #40853, #48659). Following TBST washes, membranes were incubated with horseradish peroxidase‐conjugated secondary antibodies and developed with enhanced chemiluminescence reagents. Bands were quantified using ImageJ.

### 2.20 Mitochondrial DNA Copy Number

Genomic DNA was extracted using a commercial rapid extraction kit (Zhengzhou Huigengda Biotechnology, HGD01301). mtDNA copy number was determined by qPCR targeting the D‐loop region, normalized to 18S rRNA.

### 2.21 Quantitative Real‐Time PCR (qRT‐PCR)

Total RNA was isolated using Trizol (Thermo Fisher). cDNA synthesis and qRT‐PCR were performed using HiScript III and SYBR Green kits (Vazyme). Relative gene expression was calculated by the 2^−ΔΔCt^ method.

### 2.22 Statistical Analysis

Statistical analyses were performed using SPSS 22.0 and GraphPad Prism 9. Data are presented as mean ± SD. Normality and homogeneity of variance were assessed using the Shapiro–Wilk and Levene's tests, respectively. For two‐group comparisons, Student's *t*‐test was applied when assumptions were met; otherwise, the Mann–Whitney U test was used. For multiple groups, one‐way ANOVA with Tukey's post hoc test or corresponding non‐parametric tests were performed based on data distribution. Statistical significance was defined as *p* < 0.05.

### 2.23 Data Availability

All data supporting the findings of this study are available within the paper and its Supplementary Information. RNA‐seq data reported in this paper have been deposited in the NCBI SRA database (BioProject PRJNA1293715).

## 3 Results

### 3.1 Upregulated CYP2E1 Activity Links to Obesity Severity and Adipocyte Dysfunction in HFD‐Induced Obese Mice and in vitro Models

**FIGURE 1:**
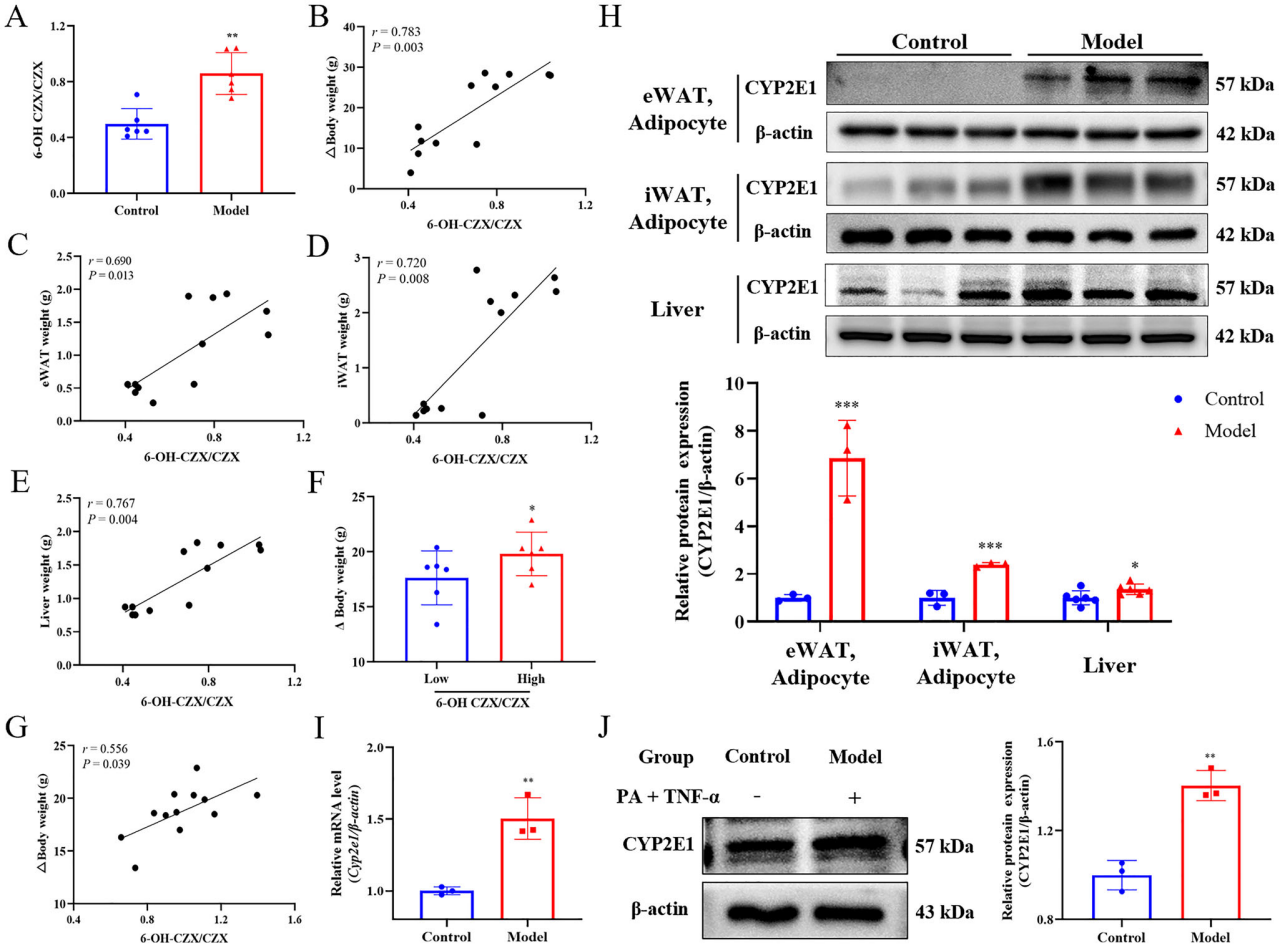
CYP2E1 is upregulated in HFD‐induced obese mice and in vitro adipocyte models. (A–G) CYP2E1 activity is elevated and positively correlates with obesity severity in HFD‐fed mice. (A) CYP2E1 activity in control versus model mice (*n* = 6). (B–E) Correlations between CYP2E1 activity and body weight gain (B), eWAT weight (C), iWAT weight (D), and liver weight (E). (F) Body weight gain differs significantly between mice stratified into high‐ and low‐activity groups based on the median baseline CYP2E1 activity (*n* = 12). (G) Correlation between innate CYP2E1 activity and weight gain. (H) CYP2E1 protein levels in primary adipocytes isolated from eWAT and iWAT (*n* = 3), and in liver tissue (*n* = 6). (I, J) CYP2E1 mRNA and protein expression in 3T3‐L1 adipocytes treated with PA (400 µm) and TNF‐α (10 ng/mL) for 48 h (*n* = 3). Data are presented as the mean ± SD, ^*^ *p* < 0.05, ^**^ *p* < 0.01, ^***^ *p* < 0.001 versus the control group; ^*^ *p* < 0.05 versus the low group.

To investigate the association between CYP2E1 activity and obesity, in vivo CYP2E1 activity was measured via plasma 6‐OH CZX/CZX ratios by HPLC. After 12 weeks of HFD feeding, CYP2E1 activity was significantly elevated in obese mice (Figure 1A), positively correlating with body weight gain (Figure 1B), eWAT weight (Figure 1C), iWAT weight (Figure 1D), and liver weight (Figure 1E).

Baseline CYP2E1 activity measured prior to HFD was stratified into high‐ and low‐activity groups based on the median value. Mice in the high‐activity group gained significantly more weight under HFD feeding compared to the low‐activity group (Figure 1F).

Western blot analysis revealed robust upregulation of CYP2E1 protein in mature adipocytes isolated from eWAT (5.9‐fold) and iWAT (1.4‐fold), and moderately in liver tissue (0.4‐fold) in HFD‐fed mice compared to controls (Figure 1H). in vitro, PA and TNF‐α co‐treatment significantly increased CYP2E1 mRNA and protein levels in adipocytes (Figure 1I,J). These data indicate a close link between elevated CYP2E1 activity and obesity‐associated adipocyte dysfunction.

### 3.2 Q11 Dose‐Dependently Attenuates HFD‐Induced Obesity without Affecting Food Intake

**FIGURE 2:**
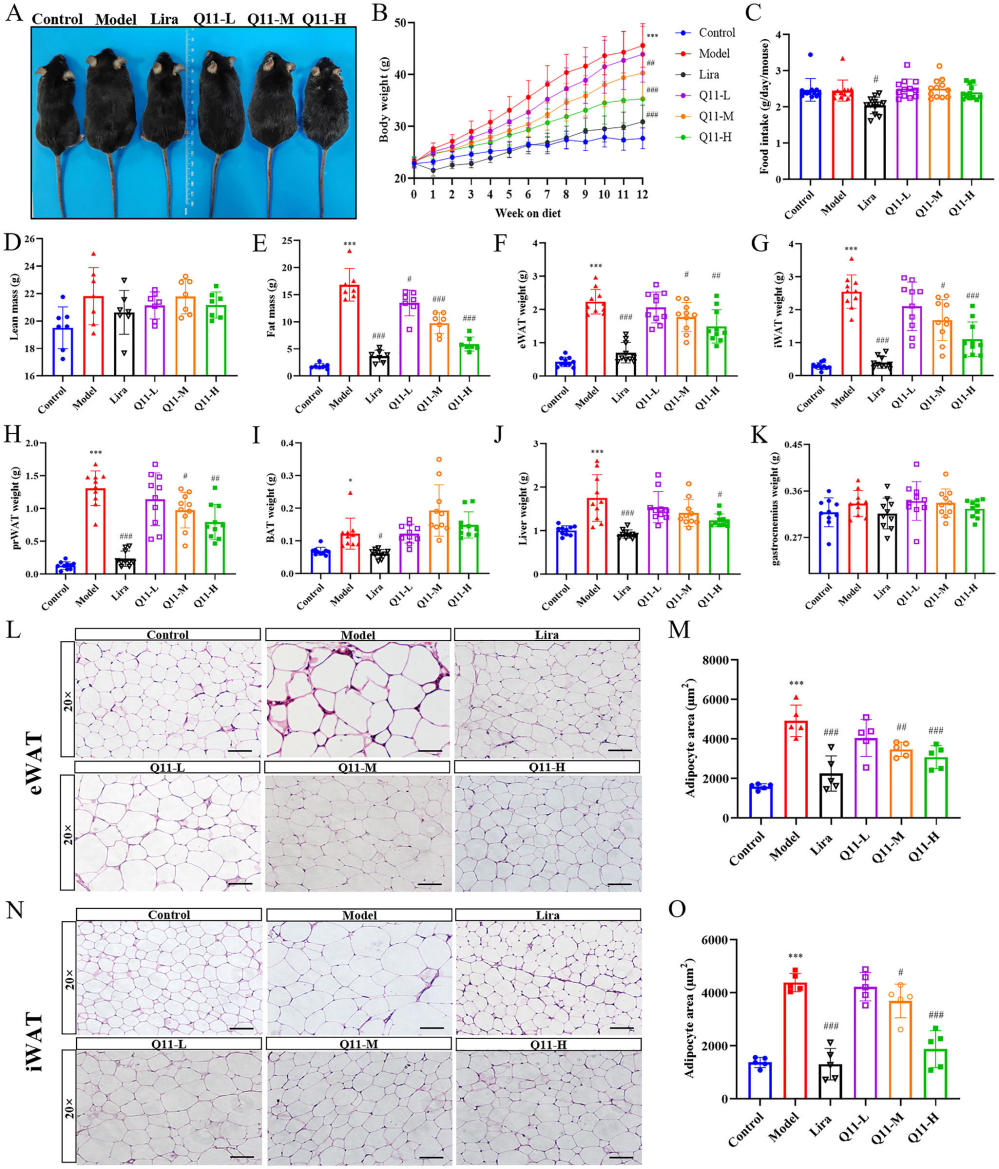
Q11 attenuates body weight gain and fat accumulation in HFD‐induced obese mice. (A) Representative images of mice from each group. (B) Weekly body weight gain (*n* = 10). (C) Average daily food intake (*n* = 12). (D, E) Body composition analysis showing fat and lean mass (*n* = 7). (F–K) Weights of iWAT (F), eWAT (G), prWAT (H), BAT (I), liver (J), and gastrocnemius (K) at sacrifice. (L–O) H&E staining (L, N) and quantification of adipocyte size (M, O) in eWAT and iWAT. Scale bar, 80 µm. Data are mean ± SD. ^*^ *p* < 0.05, ^***^ *p* < 0.001 versus control group. ^#^ *p* < 0.05, ^##^ *p* < 0.01, ^###^ *p* < 0.001 versus model group.

Over 12 weeks of HFD feeding, Q11 treatment reduced body weight gain in a dose‐dependent manner. Q11‐M and Q11‐H elicited effects starting from week 2, while Q11‐L showed effects from week 5. By week 12, body weight reductions reached 31.2% with Lira, and 1.9%, 10.5%, and 21.2% for Q11‐L, Q11‐M, and Q11‐H, respectively (Figure 2A,B; Table S1). Notably, Q11 did not alter food intake, indicating its anti‐obesity effect was independent of appetite suppression (Figure 2C).

Body composition analysis showed that fat mass was decreased dose‐dependently by Q11, while lean mass remained unchanged (Figure 2D,E). At necropsy, Q11 reduced eWAT, iWAT, and perirenal WAT (prWAT) weights in a dose‐dependent manner (Figure 2F–H), without affecting brown adipose tissue (BAT) (Figure 2I). Liver weight was significantly reduced by Q11‐H (29.4%) (Figure 2J), with no impact on muscle weight (Figure 2K). Histological analysis confirmed Q11‐mediated reduction in adipocyte hypertrophy in both eWAT and iWAT (Figure 2L–O).

### 3.3 Q11 Ameliorates Hepatic Steatosis in HFD‐Induced Obese Mice

**FIGURE 3:**
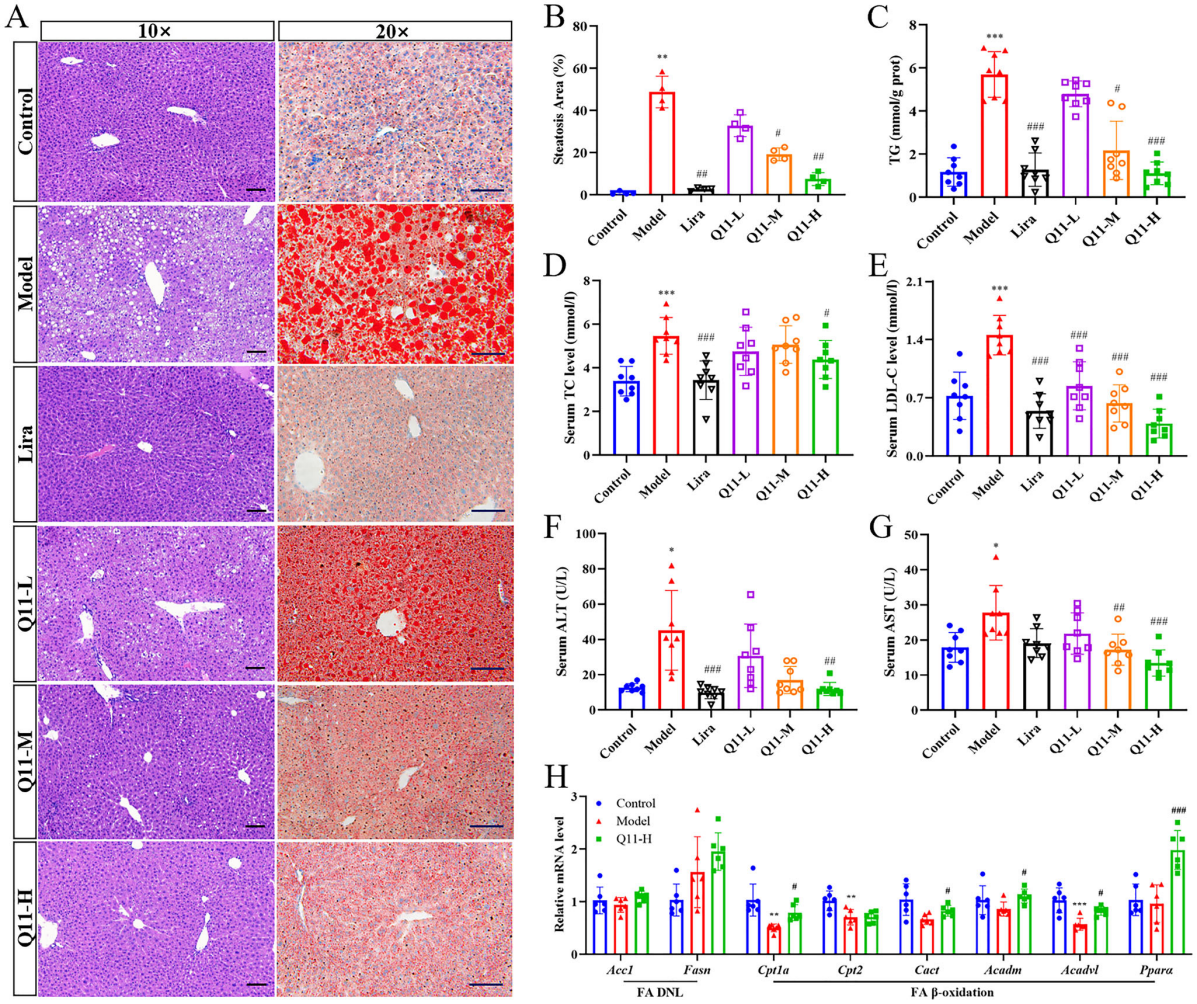
Q11 ameliorates hepatic steatosis in HFD‐induced obese mice. (A) Representative H&E and Oil Red O staining of liver sections. Scale bars, 50 or 100 µm. (B) Quantification of hepatic lipid accumulation (*n* = 4). (C) Hepatic triglyceride content (*n* = 8). (D–G) Serum levels of TC (D), LDL‐C (E), ALT (F), and AST (G) (*n* = 8). (H) Hepatic mRNA expression of fatty acid metabolism‐related genes (*n* = 6). Data as mean ± SD, ^*^ *p* < 0.05, ^**^ *p* < 0.01, ^***^ *p* < 0.001 versus control group. ^#^ *p* < 0.05, ^##^ *p* < 0.01, ^###^ *p* < 0.001 versus model group.

H&E and Oil Red O staining demonstrated marked hepatic lipid accumulation in HFD‐fed mice, which was dose‐dependently attenuated by Q11 (Figure 3A,B). Hepatic TG levels were significantly reduced by Q11‐M and Q11‐H (Figure 3C). Q11‐H also lowered serum TC and LDL‐C levels (Figure 3D,E) and improved liver injury markers (ALT, AST) (Figure 3F,G).

Mechanistically, Q11‐H significantly upregulated genes involved in β‐oxidation (*Cpt1a*, *Cact*, *Acadm*, *Acadvl*, *Pparα*), with no effect on lipogenesis genes (*Acc1*, *Fasn*) (Figure 3H). These findings suggest Q11 protects against NAFLD by enhancing hepatic fatty acid oxidation.

### 3.4 Q11 Reduces White Adipose Tissue Inflammation by Limiting Macrophage Infiltration and Adipocyte Cytokine Production

**FIGURE 4:**
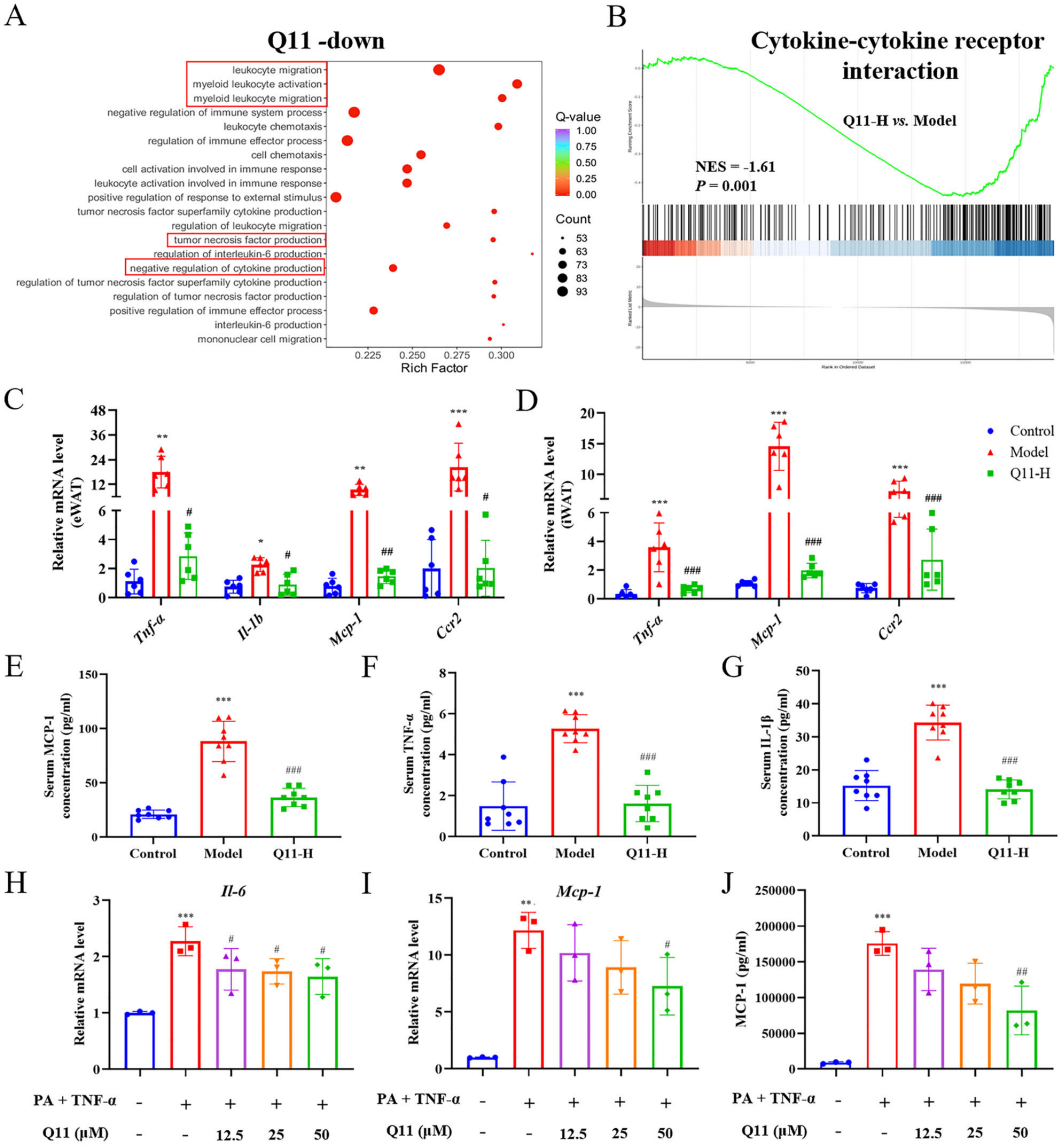
Q11 suppresses adipose tissue inflammation in obese mice and in vitro adipocyte models. (A) GO enrichment and (B) GSEA analysis showing downregulation of inflammatory pathways in eWAT of Q11‐treated mice. (C, D) Inflammatory gene expression in eWAT (C) and iWAT (D). (E–G) Serum levels of MCP‐1, TNF‐α, and IL‐1β (*n* = 8). (H–J) mRNA expression of IL‐6 (H) and MCP‐1 (I), and MCP‐1 secretion (J) in 3T3‐L1 adipocytes (*n* = 3). Data as mean ± SD. ^*^ *p* < 0.05, ^**^ *p* < 0.01, ^***^ *p* < 0.001 versus control group. ^#^ *p* < 0.05, ^##^ *p* < 0.01, ^###^ *p* < 0.001 versus model group.

To further elucidate the mechanisms underlying the anti‐obesity effects of Q11 in HFD‐fed mice, we performed transcriptomic analysis of eWAT. Compared to the model group, Q11 treatment downregulated 1824 and upregulated 1102 genes (Figure S2). Gene Ontology (GO) and Gene Set Enrichment Analysis (GSEA) indicated that Q11 predominantly suppressed inflammation‐related pathways (Figure 4A,B).

qPCR confirmed that Q11 significantly reduced pro‐inflammatory gene expression (*Tnf‐α*, *Il‐1β*, *Mcp‐1*, *Ccr2*) in eWAT and iWAT (Figure 4C,D). Serum MCP‐1, TNF‐α, and IL‐1β levels were also decreased by Q11‐H (Figure 4E–G). in vitro studies further confirmed these effects: Q11 treatment significantly reduced IL‐6 and MCP‐1 expression and lowered MCP‐1 secretion (Figure 4H–J).

### 3.5 Q11 Enhances Energy Expenditure in HFD‐Induced Obese Mice

**FIGURE 5:**
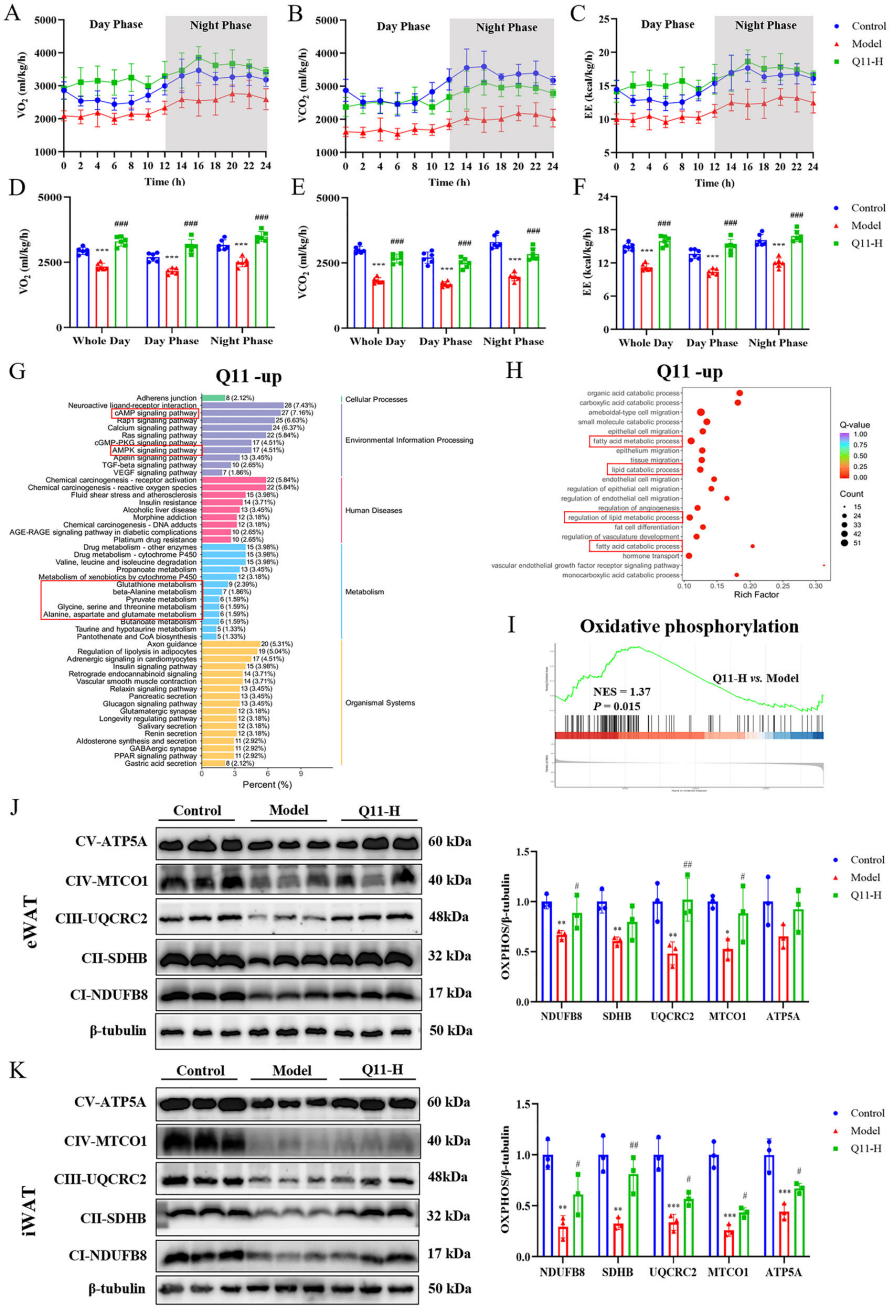
Q11 enhances energy expenditure in HFD‐induced obese mice. (A–C) Oxygen consumption (VO_2_), carbon dioxide production (VCO_2_), and energy expenditure (EE) over 24 h. (D–F) Average VO_2_, VCO_2_, and EE during light and dark cycles (*n* = 6). (G–I) KEGG, GO, and GSEA enrichment analyses of eWAT transcriptome showing activation of mitochondrial pathways by Q11. (J, K) OXPHOS protein levels in eWAT and iWAT (*n* = 3). Data are mean ± SD. ^*^ *p* < 0.05, ^**^ *p* < 0.01, ^***^ *p* < 0.001 versus control group. ^#^ *p* < 0.05, ^##^ *p* < 0.01, ^###^ *p* < 0.001 versus model group.

Given that Q11 significantly suppressed HFD‐induced weight gain without altering food intake, we next evaluated its effects on systemic energy metabolism using indirect calorimetry. Compared with control mice, HFD‐fed mice exhibited a marked decrease in VO_2_, while Q11 administration robustly increased VO_2_ by 44.09% during the light phase and 39.23% during the dark phase relative to the model group (Figure 5A,D). Similarly, VCO_2_ was significantly reduced in obese mice but elevated by 48.06% and 44.60% in Q11‐treated mice during the light and dark phases, respectively (Figure 5B,E), suggesting enhanced oxidative metabolism. Concordantly, EE was significantly decreased in the model group but was restored by Q11 treatment, showing a 44.80% and 40.21% increase in the light and dark phases, respectively (Figure 5C,F). These findings indicate that Q11 enhances energy expenditure in obese mice, likely through promoting mitochondrial oxidative capacity.

Since adipose tissue browning contributes to energy dissipation, we examined whether Q11 promotes energy expenditure through adipocyte browning. qPCR analysis revealed that in iWAT, the expression of thermogenic markers *Ucp1* and *Prdm16* was markedly reduced in HFD‐fed mice compared with controls. Q11 treatment did not significantly restore their expression (Figure S3A,B). Similarly, in BAT, *Ucp1* and *Prdm16* expression remained comparable among the three groups (Figure S3C,D). These findings suggest that the beneficial effect of Q11 on energy expenditure is unlikely to involve adipose tissue browning or classical thermogenesis.

To elucidate the underlying mechanism, transcriptomic analysis of eWAT revealed that upregulated genes in Q11‐treated mice were significantly enriched in mitochondrial metabolism‐related pathways, including the cAMP and AMPK signaling pathways, as well as fatty acid, pyruvate, and amino acid metabolism (Figure 5G,H). GSEA further confirmed a significant upregulation of oxidative phosphorylation (OXPHOS)‐related gene sets in response to Q11. Consistent with these findings, protein levels of OXPHOS complexes I, III, and IV were markedly reduced in eWAT of HFD‐fed mice but were significantly restored following Q11 treatment (Figure 5J), with similar restoration observed in iWAT (Figure 5K).

### 3.6 Q11 Improves Mitochondrial Function and Reduces Oxidative Stress in Adipocytes

**FIGURE 6:**
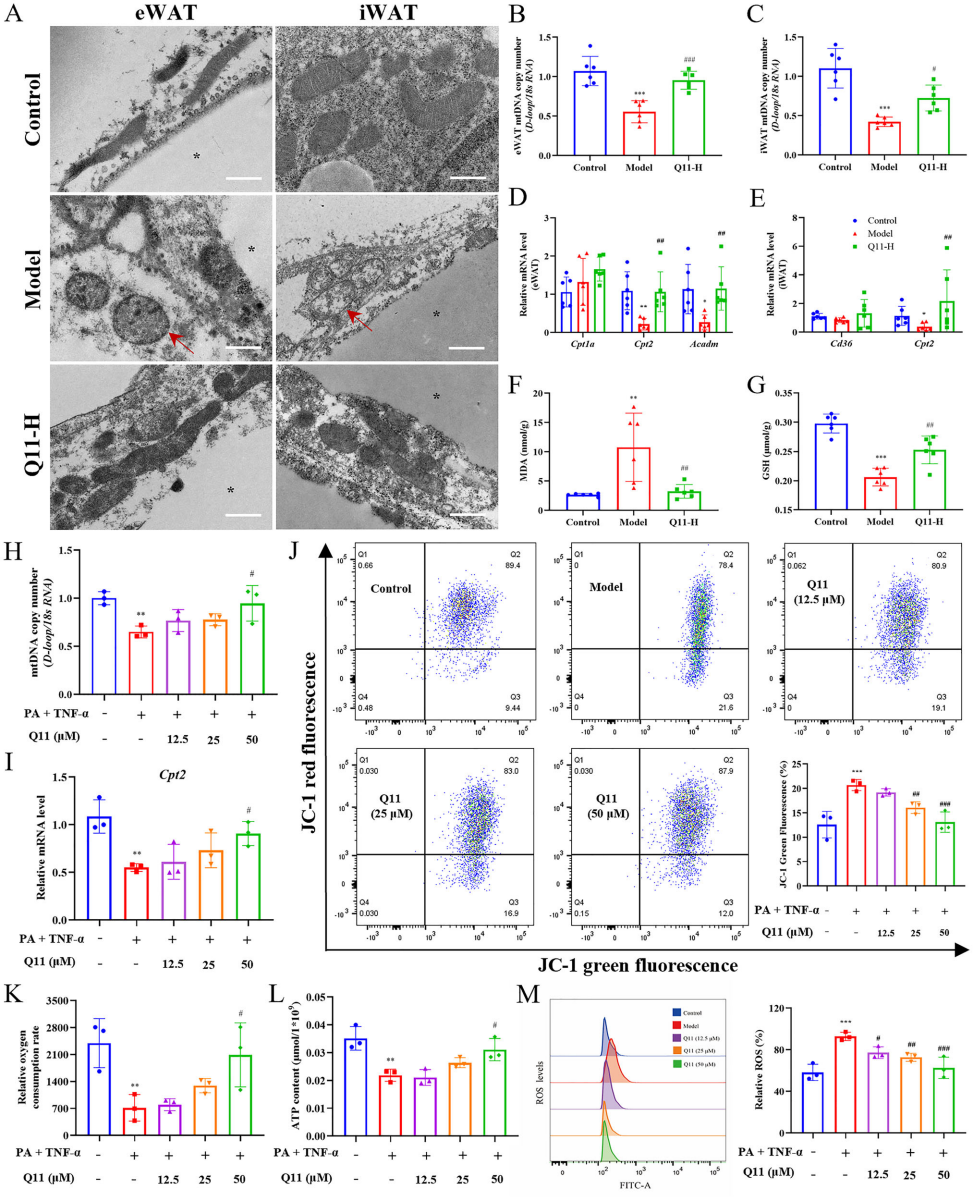
Q11 improves mitochondrial function and reduces oxidative stress in adipocytes. (A) Representative transmission electron microscopy (TEM) images of adipocytes in eWAT and iWAT. Red arrows denote representative swollen mitochondria with loss of cristae. Asterisks indicate lipid droplets. Scale bar, 1 µm. (B, C) mtDNA copy number in eWAT and iWAT. (D, E) mRNA levels of fatty acid oxidation genes. (F, G) GSH and MDA levels in eWAT. (H–M) Mitochondrial indices in 3T3‐L1 adipocytes (*n* = 3): mtDNA (H), *Cpt2* expression (I), ROS (J), mitochondrial membrane potential (ΔΨm) via JC‐1 staining (K), Oxygen consumption rate (OCR) (L), ATP content (M). Data are mean ± SD. ^*^ *p* < 0.05, ^**^ *p* < 0.01, ^***^ *p* < 0.001 versus control group. ^#^ *p* < 0.05, ^##^ *p* < 0.01, ^###^ *p* < 0.001 versus model group.

Mitochondrial integrity is crucial for adipocyte function and whole‐body energy balance. Given the strong link between mitochondrial morphology and function, we examined the effects of Q11 on mitochondrial ultrastructure in WAT by transmission electron microscopy. In eWAT from control mice, mitochondria exhibited elongated morphology and dense, well‐organized cristae. By contrast, mitochondria from HFD‐fed mice appeared swollen, with disrupted or absent cristae. Q11 treatment markedly attenuated these structural abnormalities, preserving mitochondrial shape and cristae integrity. Similar protective effects were observed in iWAT (Figure 6A).

To assess mitochondrial biogenesis, we quantified mtDNA content via *D‐loop* expression. *D‐loop* levels were significantly reduced in both eWAT and iWAT of obese mice, but were markedly upregulated by Q11‐H (Figure 6B,C). Moreover, Q11 restored the expression of key fatty acid β‐oxidation enzymes, including *Cpt2* and *Acadm*, which were downregulated in obese adipose tissue (Figure 6D,E).

Given the role of oxidative stress in obesity‐related adipose dysfunction, we measured GSH and MDA levels. Q11 treatment significantly restored GSH content and reduced MDA accumulation in eWAT, suggesting improved redox homeostasis (Figure 6F,G).

In vitro experiments confirmed these findings. PA and TNF‐α co‐treatment impaired mitochondrial biogenesis, reduced *D‐loop* and *Cpt2* expression, and disrupted ΔΨm, as evidenced by JC‐1 staining. These changes were largely reversed by Q11‐H (Figure 6H–J). Additionally, Q11 enhanced OCR and ATP production (Figure 6K,L), while reducing intracellular ROS in a dose‐dependent manner (Figure 6M). These findings support that Q11 improves mitochondrial function and mitigates oxidative stress in adipocytes under lipotoxic conditions.

### 3.7 Q11 Activates AMPK/PGC‐1α Signaling to Restore Mitochondrial Homeostasis

**FIGURE 7:**
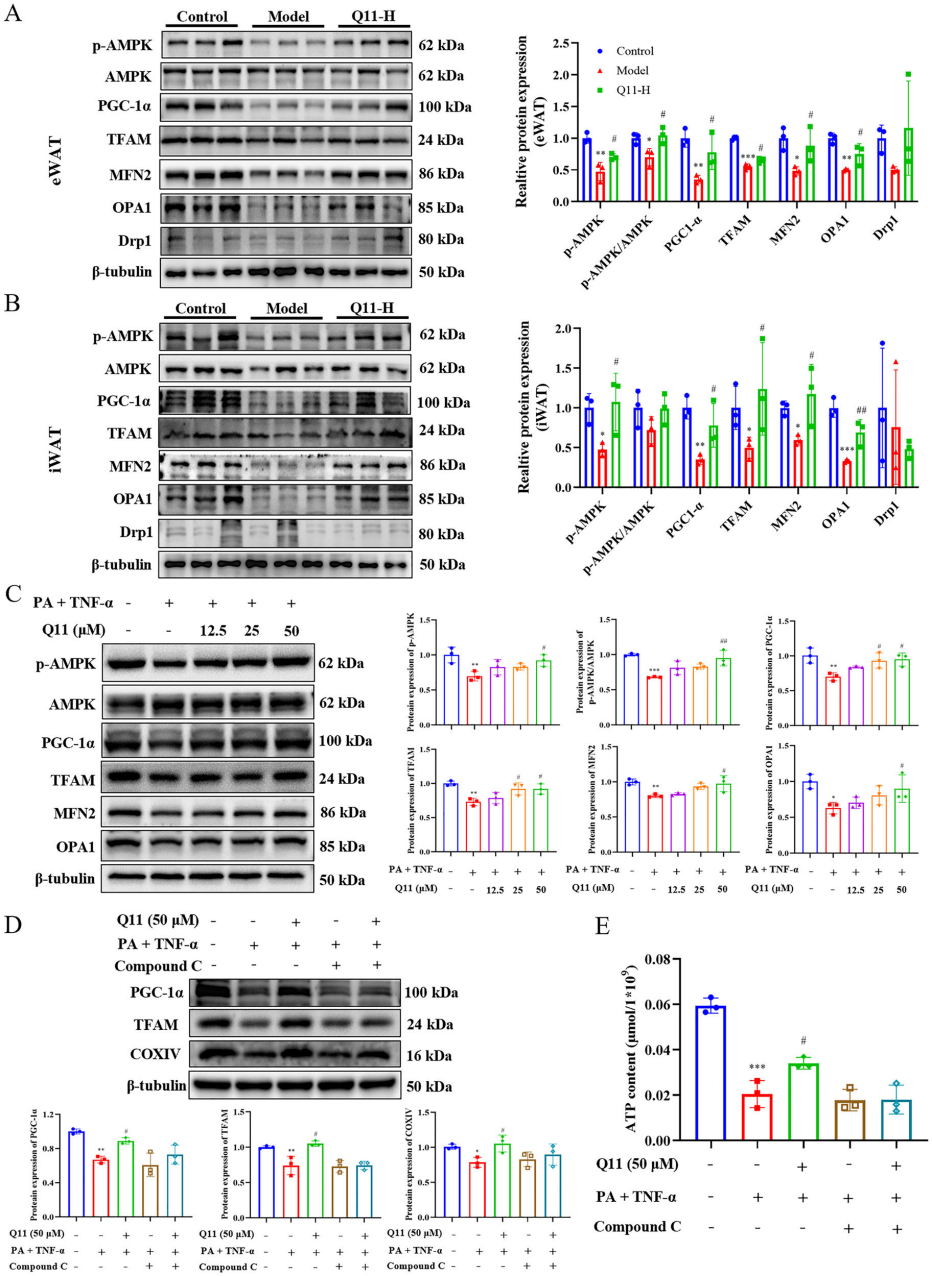
Q11 activates AMPK/PGC‐1α signaling to regulate adipocyte mitochondria. (A, B) Protein levels of AMPK, p‐AMPK, PGC‐1α, MFN2, OPA1, and DRP1 in eWAT (A) and iWAT (B) of HFD‐induced obese mice. (C) Expression of the same markers in 3T3‐L1 adipocytes treated with PA + TNF‐α with or without Q11 for 48 h. (D, E) Effects of AMPK inhibitor Compound C on Q11‐induced upregulation of PGC‐1α, TFAM, and ATP levels in vitro. The data are presented as mean ± SD, *n* = 3. ^*^ *p* < 0.05, ^**^ *p* < 0.01, ^***^ *p* < 0.001 versus control group. ^#^ *p* < 0.05, ^##^ *p* < 0.01, ^###^ *p* < 0.001 versus model group.

Transcriptomic analysis of Q11‐treated WAT suggested enrichment of the AMPK pathway. Given AMPK's central role in regulating mitochondrial biogenesis and dynamics, we examined the expression of AMPK and its downstream targets. In eWAT, HFD‐fed mice exhibited marked downregulation of phosphorylated AMPK (p‐AMPK), PGC‐1α, TFAM, and mitochondrial fusion proteins MFN2 and OPA1, while Q11‐H treatment significantly restored their expression levels (Figure 7A). These findings were mirrored in iWAT (Figure 7B).

In 3T3‐L1 adipocytes, PA and TNF‐α stimulation suppressed expression of p‐AMPK, PGC‐1α, TFAM, MFN2, and OPA1, whereas Q11 significantly upregulated all of these proteins (Figure 7C). These results indicate that Q11 promotes mitochondrial biogenesis and fusion via AMPK activation.

**FIGURE 8:**
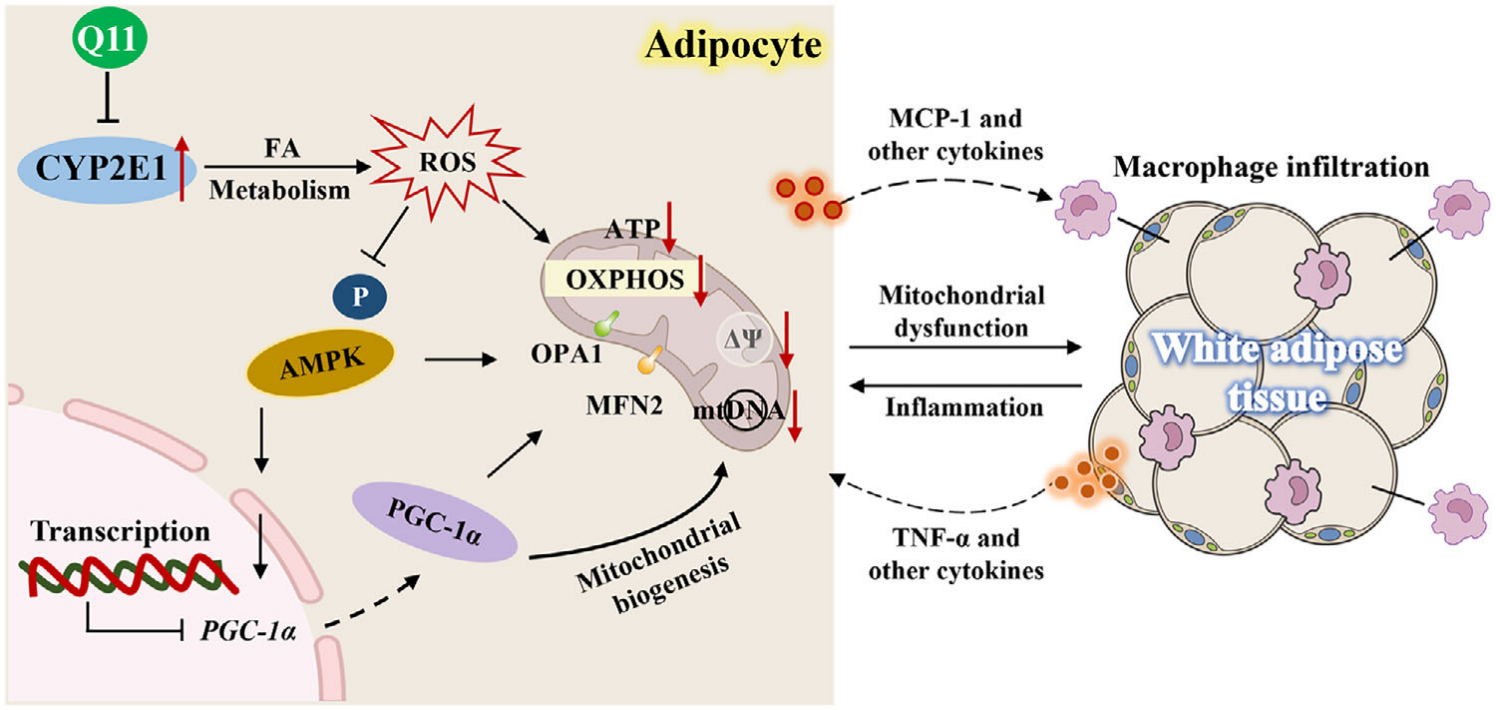
Proposed mechanism of Q11‐mediated anti‐obesity effects. Q11 alleviates obesity by improving mitochondrial function and suppressing adipose tissue inflammation. Abbreviations: ROS, Reactive oxygen species; ΔΨ, mitochondrial membrane potential; OXPHOS, Oxidative phosphorylation; AMPK, AMP‐activated protein kinase; PGC‐1α, Peroxisome proliferator‐activated receptor gamma coactivator 1‐alpha; MFN2, Mitofusin 2; OPA1, Optic atrophy 1.

To determine whether AMPK is essential for the protective effects of Q11, we co‐treated adipocytes with the AMPK inhibitor Compound C. Q11‐induced upregulation of PGC‐1α, TFAM, and COXIV was abrogated by AMPK inhibition (Figure 7D). Likewise, the increase in ATP production following Q11 treatment was suppressed by Compound C (Figure 7E), confirming an AMPK‐dependent mechanism. Taken together, Q11 exerts an anti‐obesity effect by regulating the mitochondrial function and inflammatory response of adipocytes (Figure 8).

## 4 Discussion

With the global prevalence of obesity continuing to rise, there is an urgent need to explore novel therapeutic targets and pharmacological strategies, despite the proven efficacy of GLP‐1 receptor agonists in body weight management [36]. Adverse effects and poor tolerance in certain populations often limit the utility of these agents [8, 9, 10]. Obesity is characterized by chronic, low‐grade inflammation within WAT, leading to oxidative stress, mitochondrial dysfunction, and metabolic imbalance [1, 37, 38]. In this study, we systematically uncover the critical role of CYP2E1 in the onset and progression of obesity and demonstrate that Q11, a newly developed selective CYP2E1 inhibitor, effectively prevents HFD‐induced obesity while ameliorating adipocyte dysfunction, highlighting its translational potential.

WAT inflammation is a central driver of metabolic derangement in obesity. Obese adipocytes exhibit elevated expression of pro‐inflammatory cytokines such as MCP‐1, IL‐6, and TNF‐α, which further impair metabolic function [39, 40, 41]. Concurrently, reduced mitochondrial content, impaired function, and disrupted dynamics are hallmarks of obese WAT, contributing to excessive ROS production and compromised energy homeostasis [42, 43, 44]. Existing therapies targeting inflammation and mitochondrial dysfunction remain insufficient, suggesting the involvement of yet unidentified regulatory targets [45, 46, 47, 48].

CYP2E1, a cytochrome P450 enzyme involved in the metabolism of fatty acids and xenobiotics, has gained attention for its role in oxidative stress and inflammation [15, 49, 50, 51]. Our prior studies demonstrated upregulated CYP2E1 across multiple inflammation‐associated diseases, and genetic deletion of *Cyp2e1* conferred protection. Extending these observations, we show that CYP2E1 activity correlates strongly with body‐weight gain, adiposity, and hepatic lipid accumulation in HFD‐fed wild‐type mice. Notably, baseline CYP2E1 activity predicted diet‐induced weight gain, suggesting its potential as both a biomarker and a mechanistic driver. Elevated CYP2E1 expression in mature adipocytes further implicates its regulatory role in adipose dysfunction, supporting the hypothesis that pharmacological inhibition of CYP2E1 may serve as an effective anti‐obesity strategy.

Current CYP2E1 inhibitors, including disulfiram, diallyl sulfide, and phenethyl isothiocyanate, suffer from drawbacks such as poor selectivity, toxicity, and suboptimal pharmacokinetics. In contrast, Q11 demonstrates high selectivity, potency, and a favorable safety profile, consistent with the i*n vivo* safety observed for both genetic ablation and long‐term pharmacological inhibition of CYP2E1 [22]. In a purified 60% HFD‐induced obesity model that recapitulates human disease features, 12‐week Q11 treatment significantly reduced body weight gain without affecting food intake, with the Q11‐H achieving approximately 20% weight reduction. Distinct from the appetite‐suppressing positive control Lira, Q11 lowers body weight independent of energy intake while concurrently ameliorating hepatic steatosis and systemic inflammation, underscoring its multifactorial therapeutic potential.

Mechanistically, adipocyte dysfunction in obesity manifests as impaired mitochondrial biogenesis, diminished ATP production, and elevated ROS [41, 42, 52, 53, 54, 55]. Q11 counteracted these deficits by enhancing mitochondrial oxygen consumption, restoring membrane potential, and upregulating key biogenesis regulators such as PGC‐1α and TFAM. Concurrently, Q11 elevated mitochondrial fusion proteins MFN2 and OPA1, indicating improved organellar dynamics. These effects were mediated through AMPK, whose phosphorylation was promoted by Q11, leading to PGC‐1α activation—a cascade abolished by AMPK inhibition. Since ROS overproduction is known to suppress AMPK [56, 57], the amelioration of oxidative stress by Q11 likely contributes to AMPK reactivation and subsequent mitochondrial restoration.

Transcriptomic profiling of eWAT further substantiated this mechanism, revealing that Q11 concurrently suppressed inflammatory pathways and enhanced OXPHOS gene expression. We propose that these effects originate in parallel from a common upstream event—CYP2E1 inhibition and the consequent reduction in ROS. Attenuated ROS not only dampens pro‐inflammatory signaling but also relieves oxidative inhibition on mitochondrial components [25, 42, 52, 58, 59, 60, 61, 62], thereby facilitating AMPK/PGC‐1α‐driven OXPHOS expression. The resulting mitochondrial recovery subsequently reinforces redox balance, while the resolved inflammatory milieu further supports mitochondrial integrity, establishing a self‐reinforcing virtuous cycle that sustainably reverses adipocyte dysfunction.

Collectively, the restoration of mitochondrial homeostasis in adipocytes attenuates ROS‐driven inflammatory signals and improves endocrine function. These cellular improvements likely translate to systemic metabolic benefits, including reduced systemic inflammation, ameliorated hepatic steatosis, and overall energy balance, highlighting the multi‐layered therapeutic potential of CYP2E1 inhibition.

Despite these promising results, several limitations remain. The durability of Q11's metabolic benefits after treatment cessation is unknown. Additionally, WAT inflammation involves extensive immune‐adipocyte crosstalk, and whether Q11 modulates macrophage or other stromal cell populations requires further investigation. Lastly, exploring combination strategies, particularly with incretin‐based therapies, may uncover potential synergistic effects.

In conclusion, this study systematically elucidates the critical role of CYP2E1 in obesity development and identifies CYP2E1 as a promising therapeutic target. We demonstrate for the first time that Q11 significantly ameliorates HFD‐induced obesity and metabolic dysfunction by restoring adipocyte mitochondrial function and reducing inflammation via AMPK/PGC‐1α activation. These findings provide a novel theoretical and pharmacological basis for anti‐obesity strategies targeting inflammation and mitochondrial dysfunction.

## Author Contributions

Q.H.L., Q.J.H. and G.L.Y. designed the experiments and drafted the manuscript. X.H.W. conducted synthesis and optimization of Q11 series compounds. Q.J.H., G.L.Y., W.L.Y., W.X.K., and T.L.M. performed experiments in vitro and in vivo. J.L. and D.M.Y. performed RNA sequencing. Q.J.H. and G.L.Y. conducted the statistical analyses. Q.H.L., G.N., and W.Q. directed the experimental design, oversaw the development of the study concept, and reviewed and edited the manuscript. All authors reviewed the manuscript and approved the content.

## Funding

This work was supported by grants from the National Natural Science Foundation of China (Nos. 82574493, 82274008 and 82073930).

## Conflicts of Interest

The authors declare no conflicts of interest.

## Supporting information




**Supporting File**: advs73407‐sup‐0001‐SuppMat.docx.

## Data Availability

The data that support the findings of this study are available from the corresponding author upon reasonable request.
