# Supplementary material for: A Novel CYP2E1 Inhibitor, 4‐Methyl‐5‐Acetylthiazole (Q11), Alleviates Obesity Via Modulating Adipose Inflammation and Mitochondrial Dysfunction
Source: Adv Sci (Weinh). 2025 Dec 20;13(10):e15315. doi: 10.1002/advs.202515315 (PMC12915123; doi:10.1002/advs.202515315)
Supplement: Supplementary file 1 — Supporting File: advs73407‐sup‐0001‐SuppMat.docx. [file ADVS-13-e15315-s001.docx]

**SUPPORTING INFORMATION**

**A Novel CYP2E1 Inhibitor,** **1-(4-methyl-5-thiazolyl) ethanone (Q11), Alleviates Obesity via Modulating Adipose Inflammation and Mitochondrial Dysfunction**

Jinhuan Qiu^a, 1^, Liyuan Gao^a, 1^, Liyang Wang^a^, Xueke Wang^a^, Lin Jia^a^, Mengyan Deng^a^, Liming Tang^a^, Qiang Wen^a^, Na Gao^a^, Haiwei Xu^a^, Hailing Qiao^a, *^

^a^ Institute of Clinical Pharmacology, School of Basic Medical Sciences, Zhengzhou University, Zhengzhou, 450000, China

^*^ Corresponding authors: Hailing Qiao. Email: qiaohl@zzu.edu.cn.

^1^ Jinhuan Qiu and Liyuan Gao contributed equally.

|  |
| --- |
|  |
| **** |

**Figure S1:** **Chemical structure of 4-methyl-5-acetylthiazole, defined as Q11.**

|  |
| --- |

| **Table S1 Weekly body weights of HFD-induced obese mice accompanying intragastric administration with Q11 (Mean ± SD)** | | | | | | | | | | | | | | |
| --- | --- | --- | --- | --- | --- | --- | --- | --- | --- | --- | --- | --- | --- | --- |
| Group | n | 0 W | 1 W | 2 W | 3 W | 4 W | 5 W | 6 W | 7 W | 8 W | 9 W | 10 W | 11 W | 12 W |
| Control | 10 | 23.1 ± 0.9 | 23.9 ± 1.0 | 24.2 ± 1.2 | 24.8 ± 1.5 | 25.4 ± 1.4 | 25.8 ± 1.4 | 26.7 ± 1.4 | 26.6 ± 1.6 | 27.6 ± 1.7 | 27.1 ± 1.8 | 28.0 ± 2.0 | 27.6 ± 2.1 | 27.6 ± 2.0 |
| Model | 10 | 23.1 ± 1.0 | 25.6 ± 1.2^**^ | 26.8 ± 1.6^***^ | 28.6 ± 2.2^***^ | 30.4 ± 2.4^***^ | 32.6 ± 2.7^***^ | 35.0 ± 3.3^***^ | 37.5 ± 3.7^***^ | 39.6 ± 3.7^***^ | 40.7 ± 3.9^***^ | 42.8 ± 4.1^***^ | 43.6 ± 4.2^***^ | 44.8 ± 4.3^***^ |
| Lira | 10 | 23.1 ± 0.9 | 21.8 ± 1.4^###^ | 22.6 ± 0.8^###^ | 22.9 ± 0.8^###^ | 23.9 ± 0.9^###^ | 25.2 ± 1.2^###^ | 26.5 ± 1.7^###^ | 27.0 ± 1.7^###^ | 27.8 ± 2.1^###^ | 29.0 ± 2.4^###^ | 29.5 ± 2.4^###^ | 29.9 ± 2.8^###^ | 30.8 ± 3.0^###^ |
| Q11-L | 10 | 23.2 ± 0.8 | 25.1 ± 0.8 | 26.1 ± 0.3 | 27.8 ± 1.1 | 29.2 ± 1.7 | 30.9 ± 2.2^#^ | 32.7 ± 2.9^#^ | 35.1 ± 3.3 | 37.1 ± 3.8 | 38.7 ± 4.3 | 41.4 ± 4.8^#^ | 42.7 ± 5.3^#^ | 44.0 ± 5.2 |
| Q11-M | 10 | 23.1 ± 0.7 | 24.6 ± 1.1 | 25.5 ± 1.3^#^ | 26.9 ± 1.6^##^ | 28.0 ± 1.8^##^ | 29.3 ± 2.0^###^ | 30.4 ± 2.2^###^ | 32.3 ± 2.9^###^ | 34.5 ± 3.4^##^ | 35.7 ± 3.7^##^ | 37.9 ± 4.1^###^ | 39.2 ± 4.7^###^ | 40.1 ± 4.7^###^ |
| Q11-H | 10 | 23.2 ± 0.9 | 24.8 ± 1.1 | 25.4 ± 0.9^##^ | 26.2 ± 1.1^###^ | 26.9 ± 1.4^###^ | 28.3 ± 1.8^###^ | 29.3 ± 2.3^###^ | 30.7 ± 2.9^###^ | 31.9 ± 3.4^###^ | 33.1 ± 3.5^###^ | 34.5 ± 4.1^###^ | 35.0 ± 4.4^###^ | 35.3 ± 4.7^###^ |
| ^**^*P* < 0.01, ^***^*P* < 0.001 *vs.* control group; ^#^*P* < 0.05, ^##^*P* < 0.01, ^###^*P* < 0.001 *vs.* model group. Statistical difference between groups was shown by using one-way ANOVA following multiple comparisons. DIO, diet-induced obesity; Lira, liraglutide; Q11-L, Q11-low; Q11-M, Q11-middle; Q11-H, Q11-high. | | | | | | | | | | | | | | |

| 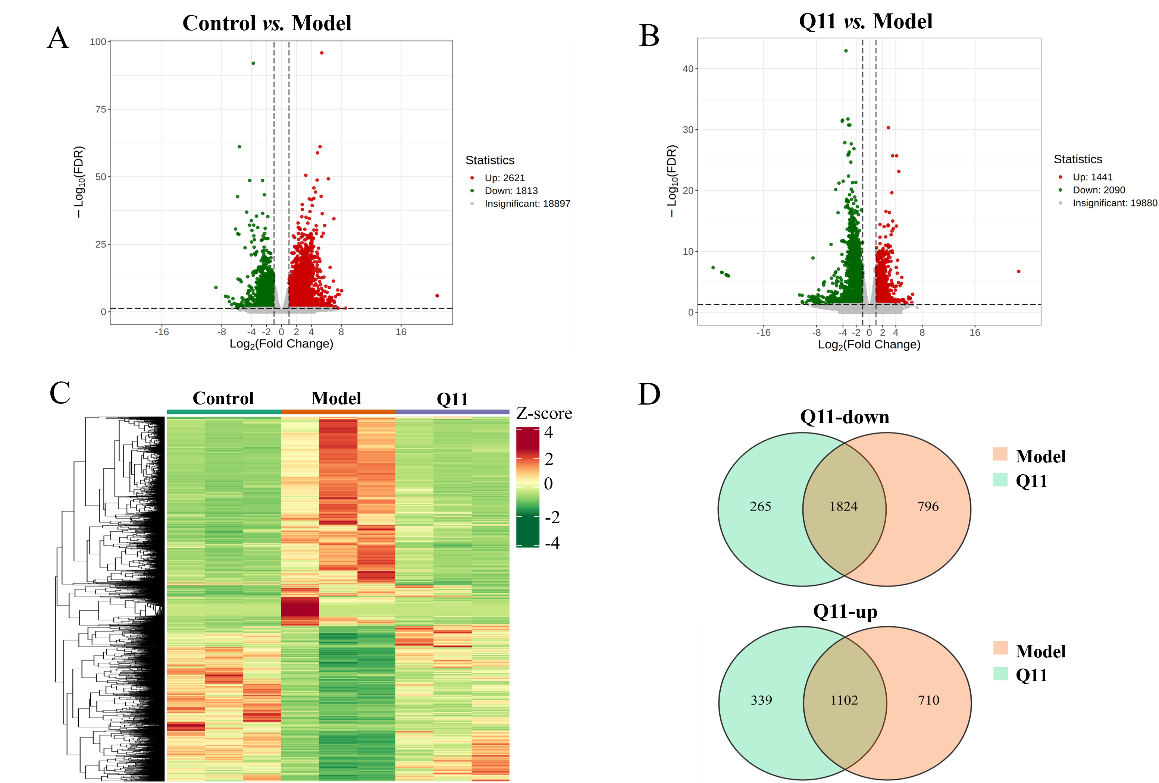 |
| --- |
|  |

**Figure S2:** **Transcriptomic analysis of differentially expressed genes in eWAT from HFD-induced obese mice treated with Q11 (60 mg/kg).** (A, B) Differential genes were shown by a volcano plot; (C) Heatmap of differentially expressed genes; (D) Common differentially expressed genes in the Q11 group identified using a Venn diagram.

| 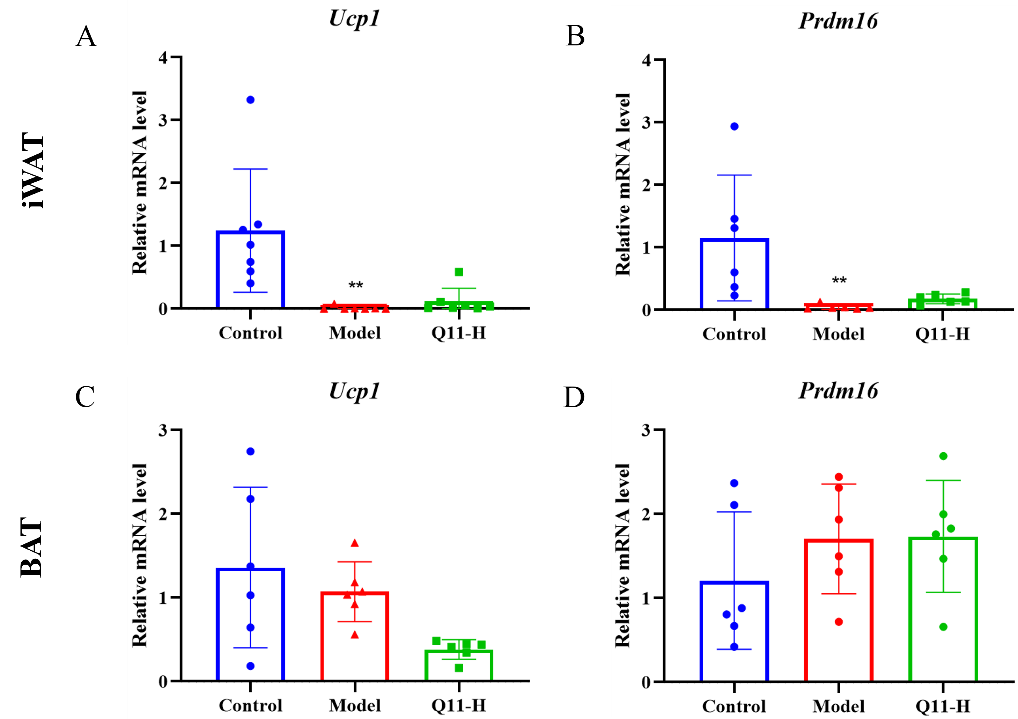 |
| --- |

**Figure S3: Effect of Q11 on thermogenic markers in iWAT and BAT of HFD-induced obese mice.** (A, B) Relative thermogenic markers (*Ucp-1* and *Prdm16*) in iWAT and (C, D) BAT were measured using real-time PCR. The data are presented as Mean ± SD, n = 6-7. Statistical difference between groups was shown by using one-way ANOVA following multiple comparisons. ***P* < 0.01 *vs.* control group.

| **Table S2 qPCR primer sequences** | |
| --- | --- |
| 引物名称 | 引物序列 |
| *β-actin* | F: GATGGTGGGAATGGGTCAGAAGG  R: TTGTAGAAGGTGTGGTGCCAGATC |
| *Cyp2e1* | F: CCAACTCTGGACTCCCTTTTAT  R: ACGCCTTGAAATAGTCACTGTA |
| *Cpt1a* | F: TGAGTGACTGGTGGGAGGAATAC  R: CAGTATGGCGTGGATGGTGTTG |
| *Cpt2* | F: TTGACCGACACTTGTTTGCTCTAC  R: AATTACCGAAGAGTTGGCGTATGG |
| *Acadm* | F: CATCATTGGCTGCTCCGTCATC  R: GTCTGAGTAGCGTCGTGGTATTCC |
| *Acadvl* | F: ACTCACTGGGCTGGGCAATG  R: CCGATTCCTGTCCTCCGTCTC |
| *Cact* | F: TACTCTGGGACCTTGGACTGTTTC  R: AAGAAGCACACGGCGAACATAG |
| *Acc1* | F: GCACTGACTGTAACCACATCTTCC  R: TCCATAGCCGACTTCCATAGCG |
| *Fasn* | F: TCCTGAAGCCGAACACCTCTG  R: GCGACAATATCCACTCCCTGAATC |
| *Pparα* | F: CCTCGGTGACTTATCCTGTGGT  R: GACATCCCGACAGAAAGGCAC |
| *Tnf-α* | F: ATGTCTCAGCCTCTTCTCATTC  R: GCTTGTCACTCGAATTTTGAGA |
| *Il-1b* | F: CACTACAGGCTCCGAGATGAACAAC  R: TGTCGTTGCTTGGTTCTCCTTGTAC |
| *Mcp-1* | F: TTTGAATGTGAAGTTGACCCGTAAATC  R: CTACAGAAGTGCTTGAGGTGGTTG |
| *Ccr2* | F: AGCCTGATCCTGCCTCTACTTGTC  R: GCCCTGTGCCTCTTCTTCTCATTC |

| **Continued Table S2 qPCR primer sequences** | |
| --- | --- |
| 引物名称 | 引物序列 |
| *Il-6* | F: CTCCCAACAGACCTGTCTATAC  R: CCATTGCACAACTCTTTTCTCA |
| *Ucp-1* | F: ACTGCCACACCTCCAGTCATT  R: CTTTGCCTCACTCAGGATTGG |
| *Prdm16* | F: GCCGTTCAAGTGCCATCTGTG  R: CCTCGTGTTCGTGCTTCTTCAG |
| *18s* | F: GCCGCTAGAGGTGAAATTCT  R: TCGGAACTACGACGGTATCT |
| *D-loop* | F: AGGCATGAAAGGACAGCA  R: TTGGCATTAAGAGGAGGG |
